# Development and validation of an expanded targeted sequencing panel for non-invasive prenatal diagnosis of sporadic skeletal dysplasia

**DOI:** 10.1186/s12920-021-01063-1

**Published:** 2021-11-17

**Authors:** Ching-Yuan Wang, Yen-An Tang, I-Wen Lee, Fong-Ming Chang, Chun-Wei Chien, Hsien-An Pan, H. Sunny Sun

**Affiliations:** 1 Institute of Molecular Medicine, College of Medicine, National Cheng Kung University, 1 University Road, Tainan, 70101 Taiwan; 2 Center for Genomic Medicine, Innovation Headquarters, National Cheng Kung University, Tainan, Taiwan; 3 FMC Fetal Medicine Center, Tainan, Taiwan; 4 AN-AN Women and Children Clinic, Tainan, Taiwan

**Keywords:** Skeletal dysplasia, Amplicon-based targeted sequencing, Noninvasive prenatal testing (NIPT), Precision medicine

## Abstract

**Background:**

Skeletal dysplasia (SD) is one of the most common inherited neonatal disorders worldwide, where the recurrent pathogenic mutations in the *FGFR2, FGFR3, COL1A1, COL1A2* and *COL2A1* genes are frequently reported in both non-lethal and lethal SD. The traditional prenatal diagnosis of SD using ultrasonography suffers from lower accuracy and performed at latter gestational stage. Therefore, it remains in desperate need of precise and accurate prenatal diagnosis of SD in early pregnancy. With the advancements of next-generation sequencing (NGS) technology and bioinformatics analysis, it is feasible to develop a NGS-based assay to detect genetic defects in association with SD in the early pregnancy.

**Methods:**

An ampliseq-based targeted sequencing panel was designed to cover 87 recurrent hotspots reported in 11 common dominant SD and run on both Ion Proton and NextSeq550 instruments. Thirty-six cell-free and 23 genomic DNAs were used for assay developed. Spike-in DNA prepared from standard sample harboring known mutation and normal sample were also employed to validate the established SD workflow. Overall performances of coverage, uniformity, and on-target rate, and the detecting limitations on percentage of fetal fraction and read depth were evaluated.

**Results:**

The established targeted-seq workflow enables a single-tube multiplex PCR for library construction and shows high amplification efficiency and robust reproducibility on both Ion Proton and NextSeq550 platforms. The workflow reaches 100% coverage and both uniformity and on-target rate are > 96%, indicating a high quality assay. Using spike-in DNA with different percentage of known *FGFR3* mutation (c.1138 G > A), the targeted-seq workflow demonstrated the ability to detect low-frequency variant of 2.5% accurately. Finally, we obtained 100% sensitivity and 100% specificity in detecting target mutations using established SD panel.

**Conclusions:**

An expanded panel for rapid and cost-effective genetic detection of SD has been developed. The established targeted-seq workflow shows high accuracy to detect both germline and low-frequency variants. In addition, the workflow is flexible to be conducted in the majority of the NGS instruments and ready for routine clinical application. Taken together, we believe the established panel provides a promising diagnostic or therapeutic strategy for prenatal genetic testing of SD in routine clinical practice.

**Supplementary Information:**

The online version contains supplementary material available at 10.1186/s12920-021-01063-1.

## Background

Skeletal dysplasia (SD) is a heterogeneous group of genetic disorders associated with various abnormalities of bones and joints. There are 461 different conditions classified into 42 groups primarily based on the clinical, radiographic, and molecular phenotypes [1]. The clinical manifestations vary in severity from mild phenotypes to severe abnormalities with perinatal mortality due to lung hypoplasia and respiratory complications [2]. Previous studies showed that the incidences of non-lethal and lethal SD are around 1 per 5,000 births [3, 4] and 0.95–1.5 per 10,000 births [5–7], respectively. The three most common SD types are thanatophoric dysplasia (around 29%) [8], osteogenesis imperfecta type 2 (14%), and achondrogenesis (9%) [9], which account for 40 to 60 percent of all lethal SD [6, 10–12]. Importantly, among the perinatal deaths of SD, 23–32% occur during the first week of life [3, 6]. The unexpected loss and highly distressing event result in a hugely psychological burden on bereaved parents. Therefore, it remains in desperate need of precise and accurate prenatal diagnosis of SD in early pregnancy.

Traditionally, the prenatal diagnosis of SD relies on ultrasonography, followed by confirmation using either invasive monogenic testing or post-delivery radiographs and autopsy. Although the ultrasound assessment of SD includes a number of features and criteria, the accuracy of routine ultrasound approach was reported as 40–60%, because the high heterogeneity in genetic defects and large phenotypic variability [13, 14]. The diagnostic confirmation of lethality was still difficult due to the lack of systematic approach [15]. In addition, the traditional genetic testing requires invasive procedures to obtain the fetal specimen such as amniocentesis and chorionic villus sampling, and the Sanger sequencing for each individual gene is costly and time-consuming [15]. Therefore, the earlier prenatal genetic diagnosis is critical issue in maternal–fetal precision medicine.

The discovery of circulating fetal cell-free DNA (cfDNA) has opened a promising opportunity for early detection of fetal genetic defects [16, 17]. Given that the fetal fraction in maternal blood is estimated to be around 4%-30% depending on the gestational age and maternal weight effects [18–20], it requires a more sensitive platform to detect the low-frequency variants in association with the fetus using maternal plasma cfDNA. Owing to the advantages of high-throughput, parallel sequencing techniques, the non-invasive prenatal testing (NIPT) has been developed and applied to identify fetal chromosomal aneuploidies using the cfDNA extracted from a blood of pregnant woman [21, 22]. Although the development is still in its infancy, the precise and flexible applications of NIPT in the detection of single-nucleotide variant (SNV) and small deletion/insertion (Indel) have been reported [23–25].

To detect low-frequency variants at single-base resolution, targeted sequencing (targeted-seq) that offers ultra-deep coverages on the genomic region of interest has been shown to function accurately and adequately [26, 27]. Furthermore, targeted-seq is cost-effectiveness in comparison with whole genome sequencing or whole-exome sequencing [28]. Two major methods, amplicon- and capture-based that utilize multiplex polymerase-chain reaction (PCR) and hybridization by probes, respectively, have been established to enrich targeted regions of interest for downstream sequencing. Previously, an amplicon-based targeted-seq for prenatally screen of *FGFR3* gene [23, 24] and a capture-based targeted-seq for screening 497 genes including several SD-associated genes [29] have been developed. Due to the restrictions by fewer mutation hotspots or lower coverage depth, it limits the application of these panels in routine clinical practice. Thus it remains in need to establish an expanded targeted sequencing panel for the detection of sporadic SD in early pregnancy.

The advantages of amplicon-based technique are the less requirement of input DNA and higher on-target rate when compared to the capture-based method, thus it is more attractive to be applied in NIPT where the fetal cfDNA is in limited amount and high accuracy is on-demand. Here, we report the development of an expanded amplicon-based targeted-seq panel for the detection of SD at ultra-depth level. This panel examines a total of 87 recurrent hotspots located in 5 most common genes (*FGFR2*, *FGFR3*, *COL1A1*, *COL1A2*, *COL2A1*) associated with 11 dominant SD, in which 5 mutations responsible for lethal SD are included. The targeted-seq workflow can be applied to genomic DNA as well as cfDNA, both achieve high sensitivity and specificity of 100%. Most importantly, the workflow is optimized to be carried out on both Illumina and Ion Torrent platforms, which account for more than 90% of instrument marketplace. Collectively, we streamline the targeted-seq workflow of two different systems and confirm its performance in clinical practice. The workflow provides timely detection of SD at early pregnancy and shows the true value of prenatal precision medicine.

## Methods

### Human subjects

A total of 59 DNA specimen, including 36 cell-free (cf) and 23 genomic (g) DNA, were collected from 6 families and 31 pregnant women (Table 1). Among them, family 1 and 2 are with fetus that were diagnosed with suspected dwarfism and osteogenesis imperfecta by ultrasound examination, respectively, while the parents were all asymptomatic. All the others were cases collected during regular pregnancy examination to screen genetic defect at early gestational age and have been followed through the gestation period until baby was delivered. Written and signed informed consent was obtained from all subjects of this study and approved by the Institutional Review Board (IRB) of the National Cheng Kung University.

**Table 1:** Summary of clinical samples used in this study

Category	Source	Sample Type^a^	Age (years)/Gestational age (weeks)	Ethnicity
Family1	Paternal blood Maternal blood Fetus’s umbilical cord	gDNA gDNA gDNA	NA 27 17W2 (terminated)	East Asian
Family2	Paternal blood Maternal blood Fetus’s umbilical cord Maternal blood	gDNA gDNA gDNA cfDNA	37 28 21W1 (terminated) 21W1	East Asian
Family3	Paternal blood Maternal blood	gDNA gDNA cfDNA	34 32 14W1	East Asian
Family4	Paternal blood Maternal blood	gDNA gDNA cfDNA	NA 38 12W4	East Asian
Family5	Paternal blood Maternal blood	gDNA gDNA cfDNA	37 32 15W4	East Asian
Family6	Paternal blood Maternal blood	gDNA gDNA cfDNA	35 33 NA	East Asian
Individual	Maternal blood	gDNA cfDNA	36 14W1	East Asian
Individual	Maternal blood	gDNA cfDNA	29 16W0	East Asian
Individual	Maternal blood	gDNA cfDNA	31 11W4	East Asian
Individual	Maternal blood	gDNA cfDNA	28 21W1	East Asian
Individual	Maternal blood	gDNA cfDNA	41 12W4	East Asian
Individual	Maternal blood	gDNA cfDNA	35 14W	East Asian
Individual	Maternal blood	gDNA cfDNA	33 13W2	East Asian
Individual	Maternal blood	gDNA cfDNA	33 16W0	East Asian
Individual	Maternal blood	gDNA cfDNA	27 21W1	East Asian
Individual	Maternal blood	cfDNA	13W	East Asian
Individual	Maternal blood	cfDNA	12W4	East Asian
Individual	Maternal blood	cfDNA	17W1	East Asian
Individual	Maternal blood	cfDNA	13W	East Asian
Individual	Maternal blood	cfDNA	13W2	East Asian
Individual	Maternal blood	cfDNA	18W7	East Asian
Individual	Maternal blood	cfDNA	12W2	East Asian
Individual	Maternal blood	cfDNA	12W6	East Asian
Individual	Maternal blood	cfDNA	11W2	East Asian
Individual	Maternal blood	cfDNA	12W6	East Asian
Individual	Maternal blood	cfDNA	13W1	East Asian
Individual	Maternal blood	cfDNA	16W3	Southeast Asian
Individual	Maternal blood	cfDNA	11W3	East Asian
Individual	Maternal blood	cfDNA	13W0	East Asian
Individual	Maternal blood	cfDNA	11W	East Asian
Individual	Maternal blood	cfDNA	14W0	East Asian
Individual	Maternal blood	cfDNA	11W3	East Asian
Individual	Maternal blood	cfDNA	16W1	East Asian
Individual	Maternal blood	cfDNA	12W1	Caucasian
Individual	Maternal blood	cfDNA	15W6	East Asian
Individual	Maternal blood	cfDNA	11W0	East Asian
Individual	Maternal blood	cfDNA	14W2	East Asian

^a^ gDNA, genomic DNA; cfDNA, cell-free DNA

### Genomic DNA collection and processing

Roughly 10 ml of peripheral blood was drawn from all participants and in two cases of Family l and Family 2, umbilical tissue was obtained from the deceased fetus. The buffy coat was separated from peripheral blood through centrifugation at 3000 rpm for 10 min at room temperature. DNA of umbilical cord and buffy coat were isolated by QIAamp DNA Mini Kit (QIAGEN, Hilden, Germany) according to the manufacturer’s protocol. The genomic DNA was stored at -20 °C for long term storage.

The standard DNA “NA11316” harboring homozygous *FGFR3* mutation (c.1138 G > A) was purchased from Coriell Institute (Coriell Institute, Camden, New Jersey, USA). To generate the spike-in DNA, NA11316 DNA was mixed with normal maternal gDNA (from Family 2) in different percentage (2.5%, 5%, 10%) to mimic different fetal DNA fractions in the maternal plasma.

### Plasma preparation and cfDNA extraction

Maternal plasma was obtained by a two-step centrifugation process. In the first step, the maternal peripheral blood was separated by centrifugation of the collection tube at 2,000 g for 15 min at room temperature and the supernatant was transferred to a 1.5 ml centrifuge tube. The sample was then centrifuged at 14,000*g* for 10 min at room temperature and the clear plasma was stored at − 80 °C until further processing.

To isolate cfDNA, maternal plasma was centrifuged at 16,000*g* for 5 min at room temperature, followed by cfDNA extraction using QIAamp Circulating Nucleic Acid Kit (QIAGEN) according to the manufacturer’s protocol. The quality and quantity of cfDNA were determined by LabChip GX Nucleic Acid Analyzer (PerkinElmer, Waltham, Massachusetts, USA) and Qubit Fluorometer (Thermo Fisher Scientific, Waltham, Massachusetts, USA), respectively. The cfDNA was stored at − 20 °C until further process.

### Design primer sets for amplicon-based targeted sequencing

To develop an expanded panel to facilitate the detection of SD, we focus on five most relevant genes (*FGFR2*, *FGFR3*, *COL1A1*, *COL1A2* and *COL2A1*) which cover 11 common clinical conditions with dominant inheritance [3, 8, 30–33]. The clinical conditions include thanatophoric dysplasia, type I-II (TD1-2; OMIM #187600 and # 187601), achondroplasia (ACH; OMIM #100800), osteogenesis imperfecta, type I- IV (OI1-4; OMIM #166200, #166210, #259420, #166220), achondrogenesis, type II (ACG2; OMIM #200610), Apert syndrome (OMIM #101200), Crouzon syndrome (OMIM #123500), and Pfeiffer syndrome (OMIM #101600).

We selected 87 recurrent mutation hotspots that comprises 82 SNVs, 2 multinucleotide variants (MNVs) and 3 indels (Additional file 1: Table S1) based on the information provided from the OMIM database (https://www.omim.org), GeneReviews (https://www.ncbi.nlm.nih.gov/books/NBK1116/), and ClinVar database (https://www.ncbi.nlm.nih.gov/clinvar/). The gene panel covers literature-supported recurrent mutations for 11 common SD clinical conditions at various frequencies: TD1 (~ 3.5%) [34], TD2 (> 99%) [35], ACH (> 99%) [34, 36, 37], OI (> 68%) [38–40], ACG2 (> 28%) [34, 41], Apert syndrome (~ 99%) [32, 42, 43], Crouzon syndrome (> 39%) [32, 44, 45], Crouzon syndrome w/acanthosis nigricans (100%) [32], and Pfeiffer syndrome (> 63%) [32, 46, 47]. We applied Ion Ampliseq Designer (version 6.1.3, Thermo Fisher Scientific) to design primers and outputted a set of 51 amplicons in one single-tube multiplexing specification (Additional file 1: Table S1).

### Library construction and amplicon sequencing

**Fig. 1:**
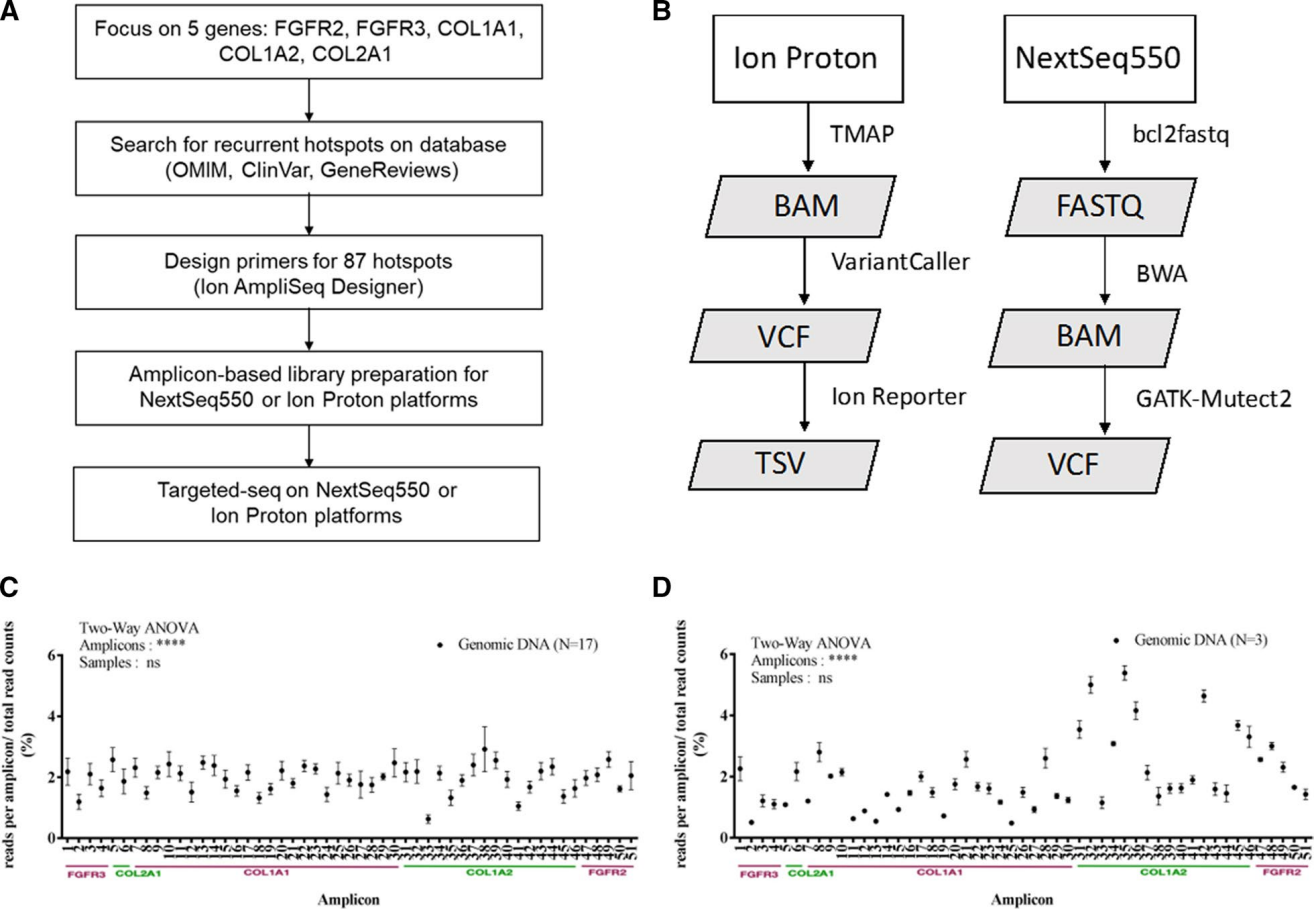
Establishment of amplicon-based targeted sequencing (targeted-seq) for skeletal dysplasia. **A** The workflow for running a 5-gene panel (87 mutation hotspots) to detect skeletal dysplasia. **B** Two bioinformatics pipelines developed for analyzing sequencing reads on Ion Proton and NextSeq550. **C**, **D** The ratio of read count of each amplicon to total reads count was plotted across 51 amplicons. The read depth was extracted from BAM file of (**C**) Ion Proton (n = 17) or (**D**) NextSeq550 (n = 3). The gene name and amplicon number are indicated (*X*-axis). *P*-values were calculated by two-way ANOVA across amplicons and samples

The amplicon libraries were constructed by Ion AmpliSeq™ Library Kit 2.0 (Thermo Fisher Scientific) according to the manufacturer’s protocol, and sequenced on Ion Proton (Thermo Fisher Scientific) or NextSeq550 instruments (Illumina, San Diego, California, USA). Briefly, approximately 10 ng of gDNA, or 1 ng of cfDNA were amplified using the 51 pairs of pooled primers in a single-tube multiplex PCR setting. The amplified libraries were then subjected to partial digestion, barcode ligation, purification, and run on Ion Proton sequencer (Fig. 1A). To modify the experimental procedure for the NextSeq550 platform, QIAseq Adapter (QIAGEN) was used for the adaptor ligation and followed by one-cycle PCR to join the adaptor and amplicon. Library concentration was determined by Ion Library TaqMan™ Quantitation Kit (Thermo Fisher Scientific) and KAPA Library Quantification Kit Illumina® Platforms (KAPA Biosystems, Wilmington, MA, USA) for Ion Proton and NextSeq550, respectively. As different barcode and index systems were used, the sizes of amplicon library were around 200 bp and 250 bp for Ion Proton and NextSeq550 systems, respectively.

### Bioinformatics pipeline

After the sequencing, the low-quality bases (Q score < 20) were trimmed and the filtered reads were aligned to human reference genome (GRCh38) using the Torrent Mapping Alignment Program (TMAP, Thermo Fisher Scientific) and Burrows-Wheeler Aligner (BWA)-v0.7.17 [48] for Ion Proton and NextSeq550 platforms, respectively (Fig. 1B). The output BAM file was used to call variant by using VariantCaller (v5.0.3.5, Thermo Fisher Scientific) under default parameters. To identify low-frequency variants in association with fetal cfDNA, a widely used haplotype-based variant calling tool for the detection of somatic or low-frequency variant [49], GATK-Mutect2, was applied. Coverage, uniformity, and on-target rate were calculated by using CoverageAnalysis (v5.0.2.0,, Thermo Fisher Scientific) or Samtools-v1.6 [50]. Alternative allele frequency of 2% and 20% were set as the thresholds for calling somatic and germline variants, respectively.

### Statistics analysis

Statistical analyses, including two-way analysis of variance (ANOVA), *Pearson* correlation coefficient (*r*), Kruskal–Wallis test, and two-tailed *t*-test, were conducted by using GraphPad Prism version 7.05 for Windows (GraphPad Software, San Diego, California USA). Data were considered significant when the *P* values were < 0.05.

## Results

### Establishment of amplicon-based targeted-seq workflow

To establish amplicon-based targeted-seq workflow (Fig. 1A, B, left), 10 ng of gDNA from 17 individuals were used for library construction and sequencing on Ion Proton platform. After sequencing, the fastq data was trimmed and then mapped to the human genome. We obtained 100% coverage of each amplicon at averaged sequencing depth of 13,160x. The uniformity and on-target rate were 99.23 ± 0.44% and 96.39 ± 3.76%, respectively. Although the mean amplicon read ratio (define by read counts in each amplicon divided to total read counts per sample) of 1.96 ± 0.45% (ratio ranged from 0.63% to 2.96%) is closed to the expected ratio of 1.96%, the amplicon read distributions are significantly different across 51 amplicons due to the characteristic of primers and GC content of each amplicon (*P* < 0.0001, Fig. 1C). Nevertheless, the pattern of amplicon distribution was no difference among 17 samples (*P* > 0.9999, Fig. 1C) and the results demonstrated high reproducibility of established workflow.

Next, we aimed to generalize the workflow to be used on NextSeq550 platform (Fig. 1A, B, right). Using slightly modified procedure and under a base coverage of 3,743x, we obtained the uniformity and on-target rate were 99.14 ± 0.92%, and 97.01 ± 0.71%, respectively. The performance is comparable to the data obtained using Ion Proton. Albeit the variation of reads distribution was slightly higher using NextSeq550 (mean ratio of 1.96 ± 1.14%, ranged from 0.49% to 5.39%) than that was using Ion Proton, common features of significant differences of read distribution across 51 amplicons (*P* < 0.0001, Fig. 1D) and high reproducibility among samples (*P* > 0.9999, Fig. 1D) were observed. Collectively, these data demonstrated the high amplification efficiency and quality in the established targeted-seq workflows. Furthermore, the flexibility of running the established targeted-seq workflow on both Ion Torrent system (i.e., Ion Proton and Ion PGM) and Illumina system (i.e., HiSeq, MiSeq, and NextSeq), the instrument implemented in the majority of diagnostic laboratories, ensure the flexibility for clinical usage.

### Validate the amplicon-based targeted-seq pipeline for the detection of skeletal dysplasia

**Fig. 2:**
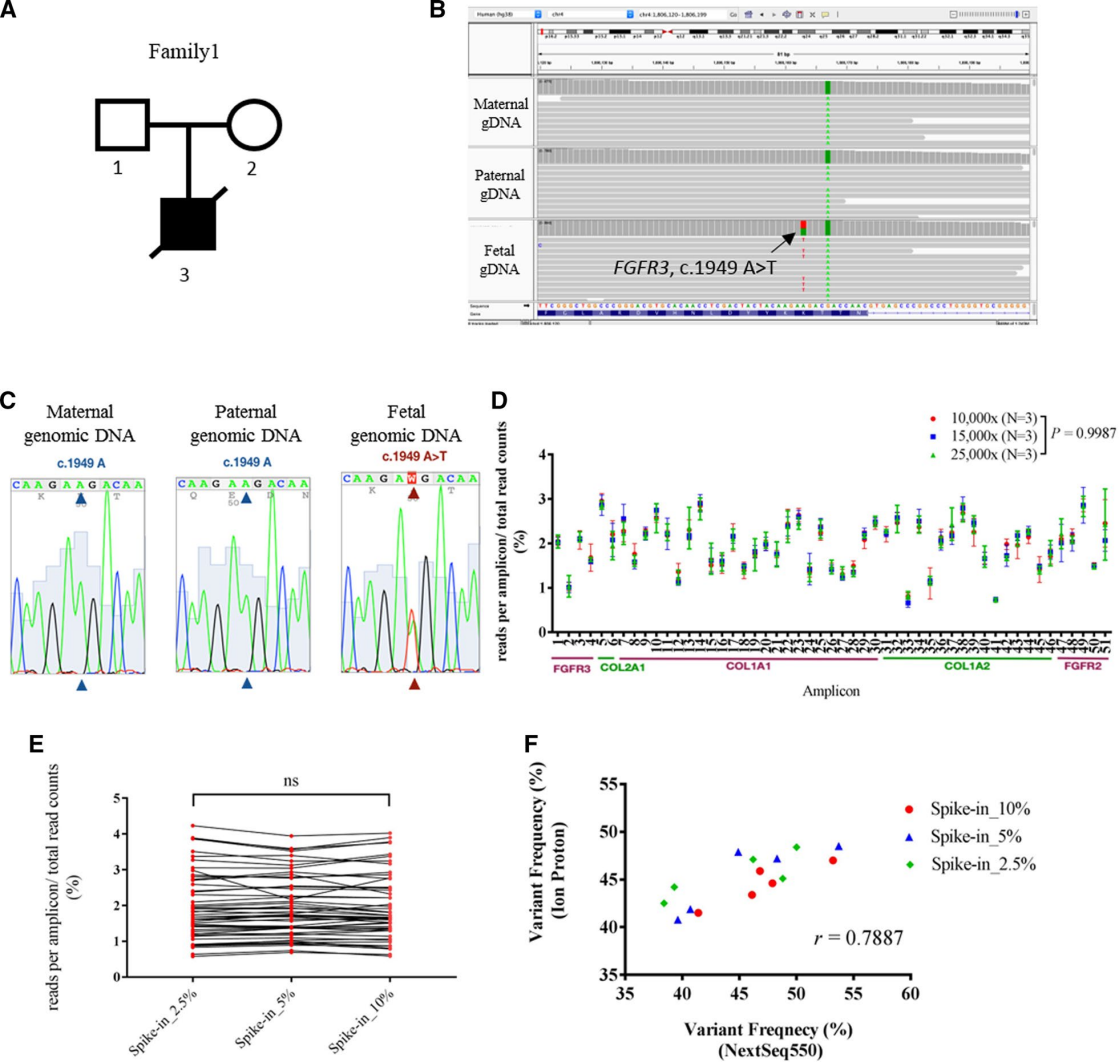
Validation of targeted-seq workflow for the detection of germline and low-frequency variants. **A** The pedigree of family 1 with abortus presenting dwarfism by ultrasound finding. **B** Visualization of reads at *FGFR3* loci (c.1949A) site by using Integrative Genomics Viewer in three samples of family 1. **C** Validation of targeted-seq results by Sanger-seq at *FGFR3* loci (c.1949A). **D** The targeted-seq was performed at expected read depth of 10,000x, 15,000x, and 25,000x on Ion Proton. The ratio of read count of each amplicon to total reads count was plotted. *P*-value was calculated by Kruskal–Wallis test. **E** The targeted-seq was performed using spike-in DNAs with *FGFR3* mutation (c. 1138G > A) at fraction of 2.5%, 5%, and 10% on NextSeq550. The ratio of read count of each amplicon to total reads count was calculated and compared. *P*-value was calculated by Kruskal–Wallis test. **F** The allele frequency of 5 SNPs obtained from Ion Proton and NextSeq550 at various spike-in fractions were compared and the correlation was calculated using *Pearson’s* correlation coefficient

We then seek to validate the targeted-seq pipeline using DNAs from a family with a fetus diagnosed as dwarfism by ultrasound images at gestational age of 21 weeks (Family 1, Fig. 2A). The targeted-seq workflow was applied to the parents’ and fetal genomic DNAs. Deep sequencing results clearly showed that the abortus carried a heterozygous mutation in *FGFR3* (c.1949 A > T, p.Lys650Met), while no mutation was detected from both parents (Fig. 2B). The identified de novo mutation was subsequently confirmed using Sanger sequencing (Fig. 2C). The mutation in *FGFR3* (c.1949 A > T) is known to cause thanatophoric dysplasia, a severe short-limb dwarfism syndrome that is usually lethal in the perinatal period (OMIM #187601), which supports the findings of targeted-seq. Taken together, the results revealed that the established targeted-seq workflow effectively identify germline mutation in association with SD.

We next challenged the capability of identifying low-frequency variant using established targeted-seq workflow. We utilized a normal maternal genomic DNA and mixed with a standard DNA sample harboring *FGFR3* single base mutation (NM_000142: c.1138G > A, p.G380R) at different percentages of 2.5%, 5%, and 10%. The spike-in DNA was used to mimic the condition in which the fetal cfDNA is mixed with maternal cfDNA at various fractions. Since the read depth has impact on the sensitivity of low-frequency variant detection, amplicon libraries were prepared and sequenced at expected coverages of 10,000x, 15,000x, and 25,000x on Ion Proton platform. We obtained the mean base coverages of 12,580x, 16,059x, and 23,904x, that were not too far away from our expectations. In spite of different read depths, the uniformities of 99.32 ± 0.21%, 98.31 ± 0.32% and 99.61 ± 0.29%, and on-target rates of 98.41 ± 0.20%, 98.43 ± 0.16% and 98.43 ± 0.20% were all similar and represented good quality results. In addition, amplicon read distributions from 12,580x (ranges from 0.71% to 2.96%, mean ratio = 1.96 ± 0.55%), 16,059x (ranges from 0.66% to 2.91%, mean ratio = 1.96 ± 0.52%), and 23,904x samples (ranges from 0.72% to 2.86%, mean ratio = 1.96 ± 0.55%) showed consistency regardless the different depths among samples (*P* > 0.99, Fig. 2D). The total depth at the spike-in *FGFR3* mutation site ranges from 5,645 to 16,835. Most importantly, we identified the spike-in *FGFR3* mutation (c.1138G > A) in all samples, even in the lowest 2.5% one with the minimum depth at 12,580x (Table 2). In addition, the *FGFR3* mutation (c.1138G > A) was detected in all spike-in samples at the values approximated to the modeled spike-in fractions (*P* = 0.11, Table 2). These data demonstrated that the workflow enables the detection of low-frequency variant accurately.

**Table 2:** Summary of *FGFR3* (c.1138G > A) variant in the spike-in DNA samples sequenced at different depths

Spike-in sample (%)	Mean depth	Total depth	Reference Allele depth (G)	Spike-in Allele depth (A)	Calculated minor-Allele frequency (%)^a^
10.0	12,580	7379	6356	1023	13.86
5.0		5645	5325	320	5.67
2.5		7080	6808	272	3.84
10.0	16,059	7852	6864	988	12.58
5.0		9043	8551	492	5.44
2.5		7990	7656	334	4.18
10.0	23,904	13,353	11,776	1577	11.81
5.0		8351	7849	502	6.01
2.5		16,835	16,182	653	3.88

^a^Paired-*t* test of spike-in fraction and calculated minor-allele frequency shows no differences between these two groups (*P* = 0.1105)

We duplicated the targeted-seq procedure with various spike-in DNA samples on NextSeq550. At mean base depth of 6,821x, we achieved uniformity and on-target rate of the samples were 98.67 ± 1.37%, and 98.03 ± 0.77%, respectively. Notably, similar amplicon read distributions among three different spike-in fractions were observed, suggesting cfDNA fraction (as indicated by various spike-in DNA fractions) has no effect on the dynamic of amplicon distribution (*P* = 0.97, Fig. 2E). As there are additional 55 single nucleotide polymorphisms (SNPs) recorded in the regions covered by the 51 amplicons (Additional file 2: Table S2) in Taiwanese population [51], we compared the results of variant calling using NextSeq550and Ion Proton systems. There were 5 SNPs identified from 3 spike-in DNA samples sequenced on two platforms. Using calculated minor-allele frequency (MAF) for these SNPs, Fig. 2F showed highly concordant results from 2 platforms (*r* = 0.79*, P* < *0.001*). Taken together, these data demonstrated the accuracy and high confidence of established targeted-seq workflows for the detection of low-frequency variants, suggesting the possible application of workflow in detecting genetic defects of SD in early pregnancy.

### The sensitivity and specificity of established targeted-seq workflow in clinical usage

Given that we have validated the targeted-seq workflow, we sought to evaluate its sensitivity and specificity using cfDNA from early pregnancy stage. Library construction and targeted-seq were performed using 36 cfDNA samples (Table 1). Among them, one sample (Family 2) was suspected to have SD at regular pregnancy examination. The fetus presented short long bones and multiple fractures using ultrasound image at 21 weeks of gestational age, which resembled a case of severe osteogenesis imperfecta (OI)-related symptoms. No mutation was identified in the cfDNA or in gDNAs from fetal umbilical cord tissue. To investigate the underlying genetic defect of the proband, we performed whole-exome sequencing and found novel compound heterozygous mutations in *CRTAP* gene consisting of SNV and deletion, suggesting the case was a rare autosomal recessive form of OI [52]. The findings supported the true negative result of cfDNA using the established targeted-seq assay. For the rest of 35 cfDNA samples, we found no mutation in the 87 hotspots from the tested SD panel. Concordant to the testing results, follow-up from these women throughout the gestation to the baby delivered showed no indication of fetal abnormality, demonstrating the accurate call for true negative results. Overall, there are 9 true positive detections (3 spike-in samples in 3 different read depths) and 36 true negative detections from our low-frequency variant tests. Although the sample size is still small, our SD targeted-seq assay reaches 100% sensitivity and 100% specificity thus it suggests the established workflow is ready for routine clinical application.

## Discussion

The diagnostics laboratories constantly strive to gain a precise and extensive genetic characterization of patients at increased efficiency, robust and cost-effectiveness. Herein, we developed an amplicon-based targeted-seq panel for the detection of 87 pathogenic mutations, including 82 SNVs, 2 MNVs and 3 indels distributing on 5 most common genes that lead to 11 autosomal dominant SDs. The targeted-seq pipeline can be applied to cell-free and genomic DNA, both of which achieve high sensitivity and specificity of 100%. Given that the turnaround time from blood sample collection to issue test report is ~ 4 days, it is feasible and cost-effective for routine screening for pregnant women at the early pregnancy. As such expanded panel, it can rule out the most common SDs from fetus and reduce the anxiety and stress in pregnancy. For the fetus presenting abnormal skeletal growth, this assay also can be a great help in genetic confirmation using genomic fetal DNA from amniocentesis or chorionic villus sampling.

The accuracy and precision are key determinants of the detection assay used in clinical diagnosis. Several studies have provided different panels to detect monogenic disorders in NIPT recently [23, 24, 29, 53]. For instance, two amplicon-based targeted-seq panels consisting of 18 and 22 hotspots in *FGFR3* genes were proposed to screen TD and ACH [23, 24], respectively. In addition, Malcher et al*.* also reported a capture-based targeted-seq procedure for screening a large panel of 497 genes in NIPT with the mean and median coverage across all sample are 267x and 222x [29]. Since the presence of fetal fraction in the maternal cfDNA can be as small as 4–5%, it needs higher coverage to precisely detect the low-frequency mutation in association with fetal cfDNA. Under the circumstance, lower overall coverage or read depth can easily lead to false negative results. Moreover, Russo et al*.* developed a amplicon-based targeted-seq for 337 mutation hotspots associated with autosomal recessive and dominate disorders for NIPT, including 42 hotspots in *FGFR2* and *FGFR3* genes [53]. However, it was a pilot study without reporting statistics of the outcome in terms of accuracy, specificity or sensitivity [53]. In the current study, we established the targeted-seq workflow that shows great performance in deep coverage, high on-target rate and uniformity. Most importantly, the workflow is able to detect variant with as low as 2.5% minor allele frequency. Because the panel is designed to detect autosomal dominant SD bearing a pathogenic mutation in heterozygous state, the minimal requirement of fetal cfDNA should be 5%, which has been set as minimal receiving criteria of fetal fraction in maternal plasma. In this study, all the clinical cfDNA are within the reporting range of fetal fraction (ranging from 5.3% to 30.9%, mean = 16.7 ± 6.7%), suggesting the reliable and accurate interpretation of the genetic testing. According to our assay development, we will run deep targeted-seq with 1 M total reads to reach at least 2000x base depths for all of 87 hotspots. Thus it provides most accurate and robust panel so far in identifying mutations for common dominant SD.

To date, several approaches have been developed for non-invasive prenatal diagnosis of a range of inherited and/or de novo transmission disorders using non-NGS or NGS-based techniques, such as droplet-digital PCR, PCR with restriction enzyme digest (PCR-RED), real-time quantitative PCR, relative dosage haplotype dosage analysis (RHDO) as well as targeted-seq. Digital PCR is useful for precise fetal genotyping by analyzing relative mutation dosages in NIPT and shows capability to detect a monogenic disorder independently of parental origin [54]. However, it requires higher amount of input DNA to reach high sensitivity and has technical difficulty of performing high-throughput in multiplex PCR, which means only for limited number of disorder. In addition, the PCR-RED is implemented in the diagnosis of *FGFR3*-associated SD in NIPT and shows high accuracy in follow-up of pregnancy outcome [23]. Unfortunately, the PCR-RED relies on subjective analysis using agarose gel electrophoresis and has an inconclusive rate of around 8% [55], suggesting the impracticable for the clinical practice. The NGS-based RHDO is applied for NIPT for monogenic disorders based on analysis of SNPs in haplotype blocks, and is able to detect complex mutations in gene loci, such as *CYP21A2* [56], and *DMD* [57]. The limitation of RHDO is the requirement of haplotype construction using parental samples, and therefore the cost is higher and prohibitive to clinical application [58]. Collectively, the above-mentioned limitations from various techniques prohibit them to be applied in NIPT for monogenic disorder detection. Intriguingly, it is reported that advanced paternal age is associated with risk of certain SDs, including TD, OI, Apert syndrome, Crouzon syndrome, and Pfeiffer syndrome [59–64], suggesting a hidden risk for transmission of deleterious variants to the offspring when aging. The paternal age-associated mutation hotspots, including *FGFR2:* c.755C > G, c.755C > T and c.758C > G [61], and *FGFR3*: c.1138G > A, c.1138G > C [64], c.1454A > G, c1948A > G and c.1949A > T [60, 62], are included in the expanded gene panel. Therefore, it further puts emphasis on the routine prenatal screen even when the parents are asymptomatic and the proposed targeted-seq workflow is useful to seize the mutations. Based on our findings, the expanded gene panel for a range of 11 SDs showed true negative results for those pregnant women with normal ultrasonography, suggesting the application is ready for screening for SD in earlier pregnancy. The turnaround time of 4 days and minimum requirement of 1 ng of cfDNA make the established targeted-seq panel an excellent choice to be offered in routine clinical practice.

It is of notice that the mutations result in rare autosomal recessive form of severe OI-related symptoms in family 2 [52] were not detected in the established panel. It is still challenging to detect mutations for autosomal recessive diseases in NIPT as the mutations are contributed from both parents thus it may present at various fractions in the mutation sites. Traditionally it requires invasive approach to obtain the fetal specimen to distinguish the origin of maternal mutation from the fetal cfDNA carries homozygous or compound heterozygous mutations [65]. Recently, Luo et al*.* reported a capture-based targeted-seq workflow that can screen for chromosomal aneuploidy of chromosomes 13, 18, and 21, microdeletions, and autosomal recessive disorders simultaneously [66]. It shows promising positive prediction rate of 100% for chromosomal aneuploidy and copy number variations, although the value for autosomal recessive disorders is still not satisfied for clinical use yet. Luo et al*.* adopts pseudo-tetraploid model instead of haplotype-based method (e.g., RHDO) to estimate the fetal genotype at specific locus, and achieved 86.4% accuracy for screening three single-gene recessive disorders [66]. Thus it is worth to develop assay and bioinformatics algorithm to enable genetic diagnosis of autosomal recessive disorders in prenatal period. Nevertheless, at current time, the combination of carrier screening for married couple and targeted-seq panel screening for pregnant women to detect mutations in association with recessive disorders in parents and dominant or *do novo* mutations in NIPT, respectively, shall provide indispensable value to reduce the deleterious effect of pathogenic mutations on human population.

## Conclusions

We established an amplicon-based targeted-seq panel that covers 87 pathogenic hotspot mutations reported in autosomal dominant inherited SD. We demonstrated the workflow is not only with high sensitivity and specificity but also shows remarkable concordance between Ion Torrent and Illumina systems. In addition, the assay requires only 1 ng of cfDNA as input material and takes minimal 4 days to accomplish entire workflow. The overall performance is considered fast and cost-effective for routine clinical practice. We believe the established panel provides a promising diagnostic or therapeutic strategy for prenatal genetic testing of SD in routine clinical practice.

## Supplementary Information

**Additional file 1: Table S1**. Summary of 87 hotspots included in this study.

**Additional file 2: Table S2**. The summary of 55 sequence variants recorded in Taiwan BioBank.

## Data Availability

All data generated or analyzed during this study are included in the main text and supplementary information files. The raw data are available upon request.

## Abbreviations

ACG2: Achondrogenesis, type II
ACH: Achondroplasia
ALT: Alternative
ALT AF: Alternative allele frequency
BWA: Burrows-Wheeler Aligner
cfDNA: Cell-free DNA
GATK: Genome Analysis Toolkit
Indel: Small deletion/insertion
MAF: Minor-allele frequency
NGS: Next-generation sequencing
NIPT: Noninvasive prenatal testing
OI1: Osteogenesis imperfecta, type I
OI2: Osteogenesis imperfecta, type II
OI3: Osteogenesis imperfecta, type III
OI4: Osteogenesis imperfecta, type IV
Sanger-seq: Sanger-sequencing
SD: Skeletal dysplasia
SNP: Single nucleotide polymorphism
SNV: Single-nucleotide variant
Targeted-seq: Targeted sequencing
TD1: Thanatophoric dysplasia, type I
TD2: Thanatophoric dysplasia, type II

## References

[1] Mortier GR, Cohn DH, Cormier-Daire V, Hall C, Krakow D, Mundlos S, Nishimura G, Robertson S, Sangiorgi L, Savarirayan R (2019). Nosology and classification of genetic skeletal disorders: 2019 revision. Am J Med Genet A.

[2] Mogayzel PJ, Marcus CL (2001). Skeletal dysplasias and their effect on the respiratory system. Paediatr Respir Rev.

[3] Orioli IM, Castilla EE, Barbosa-Neto JG (1986). The birth prevalence rates for the skeletal dysplasias. J Med Genet.

[4] Stoll C, Dott B, Roth MP, Alembik Y (1989). Birth prevalence rates of skeletal dysplasias. Clin Genet.

[5] Rasmussen SA, Bieber FR, Benacerraf BR, Lachman RS, Rimoin DL, Holmes LB (1996). Epidemiology of osteochondrodysplasias: changing trends due to advances in prenatal diagnosis. Am J Med Genet.

[6] Camera G, Mastroiacovo P (1982). Birth prevalence of skeletal dysplasias in the Italian Multicentric Monitoring System for Birth Defects. Prog Clin Biol Res.

[7] Andersen PE (1989). Prevalence of lethal osteochondrodysplasias in Denmark. Am J Med Genet.

[8] Waller DK, Correa A, Vo TM, Wang Y, Hobbs C, Langlois PH, Pearson K, Romitti PA, Shaw GM, Hecht JT (2008). The population-based prevalence of achondroplasia and thanatophoric dysplasia in selected regions of the US. Am J Med Genet A.

[9] Connor JM, Connor RA, Sweet EM, Gibson AA, Patrick WJ, McNay MB, Redford DH (1985). Lethal neonatal chondrodysplasias in the West of Scotland 1970–1983 with a description of a thanatophoric, dysplasialike, autosomal recessive disorder, Glasgow variant. Am J Med Genet.

[10] Krakow D, Alanay Y, Rimoin LP, Lin V, Wilcox WR, Lachman RS, Rimoin DL (2008). Evaluation of prenatal-onset osteochondrodysplasias by ultrasonography: a retrospective and prospective analysis. Am J Med Genet A.

[11] Tretter AE, Saunders RC, Meyers CM, Dungan JS, Grumbach K, Sun CC, Campbell AB, Wulfsberg EA (1998). Antenatal diagnosis of lethal skeletal dysplasias. Am J Med Genet.

[12] Kallen B, Knudsen LB, Mutchinick O, Mastroiacovo P, Lancaster P, Castilla E, Robert E (1993). Monitoring dominant germ cell mutations using skeletal dysplasias registered in malformation registries: an international feasibility study. Int J Epidemiol.

[13] Parilla BV, Leeth EA, Kambich MP, Chilis P, MacGregor SN (2003). Antenatal detection of skeletal dysplasias. J Ultrasound Med.

[14] Doray B, Favre R, Viville B, Langer B, Dreyfus M, Stoll C (2000). Prenatal sonographic diagnosis of skeletal dysplasias. A report of 47 cases. Ann Genet.

[15] Krakow D, Lachman RS, Rimoin DL (2009). Guidelines for the prenatal diagnosis of fetal skeletal dysplasias. Genet Med.

[16] Lo YM, Corbetta N, Chamberlain PF, Rai V, Sargent IL, Redman CW, Wainscoat JS (1997). Presence of fetal DNA in maternal plasma and serum. Lancet.

[17] Lo YM, Tein MS, Lau TK, Haines CJ, Leung TN, Poon PM, Wainscoat JS, Johnson PJ, Chang AM, Hjelm NM (1998). Quantitative analysis of fetal DNA in maternal plasma and serum: implications for noninvasive prenatal diagnosis. Am J Hum Genet.

[18] Quezada MS, Gil MM, Francisco C, Orosz G, Nicolaides KH (2015). Screening for trisomies 21, 18 and 13 by cell-free DNA analysis of maternal blood at 10–11 weeks' gestation and the combined test at 11–13 weeks. Ultrasound Obstet Gynecol.

[19] Hou Y, Yang J, Qi Y, Guo F, Peng H, Wang D, Wang Y, Luo X, Li Y, Yin A (2019). Factors affecting cell-free DNA fetal fraction: statistical analysis of 13,661 maternal plasmas for non-invasive prenatal screening. Hum Genomics.

[20] Wang E, Batey A, Struble C, Musci T, Song K, Oliphant A (2013). Gestational age and maternal weight effects on fetal cell-free DNA in maternal plasma. Prenat Diagn.

[21] Petersen AK, Cheung SW, Smith JL, Bi W, Ward PA, Peacock S, Braxton A, Van Den Veyver IB, Breman AM (2017). Positive predictive value estimates for cell-free noninvasive prenatal screening from data of a large referral genetic diagnostic laboratory. Am J Obstet Gynecol.

[22] Lo KK, Karampetsou E, Boustred C, McKay F, Mason S, Hill M, Plagnol V, Chitty LS (2016). Limited clinical utility of non-invasive prenatal testing for subchromosomal abnormalities. Am J Hum Genet.

[23] Chitty LS, Mason S, Barrett AN, McKay F, Lench N, Daley R, Jenkins LA (2015). Non-invasive prenatal diagnosis of achondroplasia and thanatophoric dysplasia: next-generation sequencing allows for a safer, more accurate, and comprehensive approach. Prenat Diagn.

[24] Terasawa S, Kato A, Nishizawa H, Kato T, Yoshizawa H, Noda Y, Miyazaki J, Ito M, Sekiya T, Fujii T (2019). Multiplex PCR in noninvasive prenatal diagnosis for FGFR3-related disorders. Congenit Anom (Kyoto).

[25] Zhang J, Li J, Saucier JB, Feng Y, Jiang Y, Sinson J, McCombs AK, Schmitt ES, Peacock S, Chen S (2019). Non-invasive prenatal sequencing for multiple Mendelian monogenic disorders using circulating cell-free fetal DNA. Nat Med.

[26] Shin H, Sa JK, Bae JS, Koo H, Jin S, Cho HJ, Choi SW, Kyoung JM, Kim JY, Seo YJ (2020). Clinical Targeted Next-Generation sequencing Panels for Detection of Somatic Variants in Gliomas. Cancer Res Treat.

[27] Kroigard AB, Thomassen M, Laenkholm AV, Kruse TA, Larsen MJ (2016). Evaluation of nine somatic variant callers for detection of somatic mutations in exome and targeted deep sequencing data. PLoS ONE.

[28] Verhoef TI, Hill M, Drury S, Mason S, Jenkins L, Morris S, Chitty LS (2016). Non-invasive prenatal diagnosis (NIPD) for single gene disorders: cost analysis of NIPD and invasive testing pathways. Prenat Diagn.

[29] Malcher C, Yamamoto GL, Burnham P, Ezquina SAM, Lourenco NCV, Balkassmi S, Antonio DSM, Hsia GSP, Gollop T, Pavanello RC (2018). Development of a comprehensive noninvasive prenatal test. Genet Mol Biol.

[30] Krakow D, Rimoin DL (2010). The skeletal dysplasias. Genet Med.

[31] Krakow D (2015). Skeletal dysplasias. Clin Perinatol.

[32] Wenger T, Miller D, Evans K. FGFR Craniosynostosis syndromes overview. In: Adam MP, Ardinger HH, Pagon RA, Wallace SE, Bean LJH, Stephens K, Amemiya A, editors. GeneReviews® [Internet]. Seattle (WA); 1998. 20301628

[33] Goncalves L, Jeanty P (1994). Fetal biometry of skeletal dysplasias: a multicentric study. J Ultrasound Med.

[34] Xue Y, Sun A, Mekikian PB, Martin J, Rimoin DL, Lachman RS, Wilcox WR (2014). FGFR3 mutation frequency in 324 cases from the International Skeletal Dysplasia Registry. Mol Genet Genomic Med.

[35] French T, Savarirayan R. Thanatophoric dysplasia. In: Adam MP, Ardinger HH, Pagon RA, Wallace SE, Bean LJH, Stephens K, Amemiya A, editors. GeneReviews® [Internet]. Seattle (WA); 2004.

[36] Legare JM. Achondroplasia. In: Adam MP, Ardinger HH, Pagon RA, Wallace SE, Bean LJH, Stephens K, Amemiya A, editors. GeneReviews® [Internet]. Seattle (WA); 1998.

[37] Ornitz DM, Legeai-Mallet L (2017). Achondroplasia: Development, pathogenesis, and therapy. Dev Dyn.

[38] Steiner RD, Basel D. COL1A1/2 Osteogenesis Imperfecta. In: Adam MP, Ardinger HH, Pagon RA, Wallace SE, Bean LJH, Stephens K, Amemiya A, editors. GeneReviews® [Internet]. Seattle (WA); 2005.

[39] Dalgleish R (1997). The human type I collagen mutation database. Nucleic Acids Res.

[40] Dalgleish R (1998). The Human Collagen Mutation Database 1998. Nucleic Acids Res.

[41] Hattapoğlu S, Durmaz MS (2018). Radiological features of achondrogenesis type 1A: case report and review of the literature. Med J Obstet Gynecol.

[42] Wenger TL, Hing AV, Evans KN. Apert Syndrome. In: Adam MP, Ardinger HH, Pagon RA, Wallace SE, Bean LJH, Stephens K, Amemiya A, editors. GeneReviews® [Internet]. Seattle (WA); 2019.

[43] Lajeunie E, Cameron R, El Ghouzzi V, de Parseval N, Journeau P, Gonzales M, Delezoide AL, Bonaventure J, Le Merrer M, Renier D (1999). Clinical variability in patients with Apert's syndrome. J Neurosurg.

[44] Al-Namnam NM, Hariri F, Thong MK, Rahman ZA (2019). Crouzon syndrome: Genetic and intervention review. J Oral Biol Craniofac Res.

[45] Lin M, Lu Y, Sui Y, Zhao N, Jin Y, Yi D, Jiang M (2019). Extremely severe scoliosis, heterotopic ossification, and osteoarthritis in a three-generation family with Crouzon syndrome carrying a mutant c.799T>C FGFR2. Mol Genet Genomic Med.

[46] Lajeunie E, Heuertz S, El Ghouzzi V, Martinovic J, Renier D, Le Merrer M, Bonaventure J (2006). Mutation screening in patients with syndromic craniosynostoses indicates that a limited number of recurrent FGFR2 mutations accounts for severe forms of Pfeiffer syndrome. Eur J Human Genet EJHG.

[47] Kan SH, Elanko N, Johnson D, Cornejo-Roldan L, Cook J, Reich EW, Tomkins S, Verloes A, Twigg SR, Rannan-Eliya S (2002). Genomic screening of fibroblast growth-factor receptor 2 reveals a wide spectrum of mutations in patients with syndromic craniosynostosis. Am J Hum Genet.

[48] Li H, Durbin R (2009). Fast and accurate short read alignment with Burrows-Wheeler transform. Bioinformatics.

[49] Cibulskis K, Lawrence MS, Carter SL, Sivachenko A, Jaffe D, Sougnez C, Gabriel S, Meyerson M, Lander ES, Getz G (2013). Sensitive detection of somatic point mutations in impure and heterogeneous cancer samples. Nat Biotechnol.

[50] Li H, Handsaker B, Wysoker A, Fennell T, Ruan J, Homer N, Marth G, Abecasis G, Durbin R (2009). Genome Project Data Processing S: The Sequence Alignment/Map format and SAMtools. Bioinformatics.

[51] Lin JC, Fan CT, Liao CC, Chen YS (2018). Taiwan Biobank: making cross-database convergence possible in the Big Data era. Gigascience.

[52] Tang YA, Wang LY, Chang CM, Lee IW, Tsai WH, Sun HS (2020). Novel Compound Heterozygous Mutations in CRTAP Cause Rare Autosomal Recessive Osteogenesis Imperfecta. Front Genet.

[53] Dello Russo C, Cesta A, Longo S, Barone MA, Cima A, Mesoraca A, Sparacino D, Viola A, Giorlandino C (2019). Validation of extensive next-generation sequencing method for monogenic disorder analysis on cell-free fetal DNA: noninvasive prenatal diagnosis. J Mol Diagn.

[54] Perlado S, Bustamante-Aragones A, Donas M, Lorda-Sanchez I, Plaza J, Rodriguez de Alba M (2016). Fetal genotyping in maternal blood by digital PCR: towards NIPD of monogenic disorders independently of parental origin. PLoS ONE.

[55] Chitty LS, Khalil A, Barrett AN, Pajkrt E, Griffin DR, Cole TJ (2013). Safe, accurate, prenatal diagnosis of thanatophoric dysplasia using ultrasound and free fetal DNA. Prenat Diagn.

[56] New MI, Tong YK, Yuen T, Jiang P, Pina C, Chan KC, Khattab A, Liao GJ, Yau M, Kim SM (2014). Noninvasive prenatal diagnosis of congenital adrenal hyperplasia using cell-free fetal DNA in maternal plasma. J Clin Endocrinol Metab.

[57] Parks M, Court S, Cleary S, Clokie S, Hewitt J, Williams D, Cole T, MacDonald F, Griffiths M, Allen S (2016). Non-invasive prenatal diagnosis of Duchenne and Becker muscular dystrophies by relative haplotype dosage. Prenat Diagn.

[58] Jenkins LA, Deans ZC, Lewis C, Allen S (2018). Delivering an accredited non-invasive prenatal diagnosis service for monogenic disorders and recommendations for best practice. Prenat Diagn.

[59] Carothers AD, McAllion SJ, Paterson CR (1986). Risk of dominant mutation in older fathers: evidence from osteogenesis imperfecta. J Med Genet.

[60] Goriely A, Hansen RM, Taylor IB, Olesen IA, Jacobsen GK, McGowan SJ, Pfeifer SP, McVean GA, Rajpert-De Meyts E, Wilkie AO (2009). Activating mutations in FGFR3 and HRAS reveal a shared genetic origin for congenital disorders and testicular tumors. Nat Genet.

[61] Goriely A, McVean GA, Rojmyr M, Ingemarsson B, Wilkie AO (2003). Evidence for selective advantage of pathogenic FGFR2 mutations in the male germ line. Science.

[62] Goriely A, Wilkie AO (2012). Paternal age effect mutations and selfish spermatogonial selection: causes and consequences for human disease. Am J Hum Genet.

[63] Lemyre E, Azouz EM, Teebi AS, Glanc P, Chen MF (1999). Bone dysplasia series. Achondroplasia, hypochondroplasia and thanatophoric dysplasia: review and update. Can Assoc Radio J.

[64] Shinde DN, Elmer DP, Calabrese P, Boulanger J, Arnheim N, Tiemann-Boege I (2013). New evidence for positive selection helps explain the paternal age effect observed in achondroplasia. Hum Mol Genet.

[65] Dan S, Yuan Y, Wang Y, Chen C, Gao C, Yu S, Liu Y, Song W, Asan, Zhu H, et al. Non-invasive prenatal diagnosis of lethal skeletal dysplasia by targeted capture sequencing of maternal plasma. PLoS ONE. 2016;11(7):e0159355. 10.1371/journal.pone.0159355 PMC4959253 27433940

[66] Luo Y, Jia B, Yan K, Liu S, Song X, Chen M, Jin F, Du Y, Wang J, Hong Y (2019). Pilot study of a novel multi-functional noninvasive prenatal test on fetus aneuploidy, copy number variation, and single-gene disorder screening. Mol Genet Genomic Med.

